# Environmentally benign liquid chromatographic method for concurrent estimation of four antihistaminic drugs applying factorial design approach

**DOI:** 10.1186/s13065-024-01117-2

**Published:** 2024-01-30

**Authors:** Rana Ghonim, Manar M. Tolba, Fawzia Ibrahim, Mohamed I. El-Awady

**Affiliations:** 1 Department of Pharmaceutical Analytical Chemistry, Faculty of Pharmacy, Mansoura University, Mansoura, 35516 Egypt; 2 Department of Pharmaceutical Chemistry, Faculty of Pharmacy, Delta University for Science and Technology, International Coastal Road, Gamasa, 11152 Egypt

**Keywords:** Rupatadine, Desloratadine, Fexofenadine, Montelukast, Green RP-HPLC-factorial design

## Abstract

In the last few decades, green analytical chemistry (GAC) has become a smart magical solution for the qualification and quantification of many drugs. In the current study, a direct, sensitive, and green RP-HPLC method was used to separate three anti-histaminic combinations rupatadine/montelukast, desloratadine/montelukast, fexofenadine/montelukast, and finally a mixture of rupatadine and its metabolite; desloratadine in less than 20 min. The developed method was optimized by a 2^3^ full factorial design to improve the chromatographic responses. The proposed method was used to analyze these antihistaminic combinations at different pharmaceutical ratios. The linearity range is from 1 to 10 µg/mL for rupatadine, desloratadine, and montelukast, while for fexofenadine from 1 to 24 µg/mL drugs. The proposed method is useful in common quality control analysis of the investigated quaternary combinations because of its non-toxic and eco-friendly effects on the environment and human beings. The proposed procedure was thoroughly validated in accordance with ICH guidelines and was revealed to be accurate, reproducible, and selective. The developed methods were compared with a reported reference comparison method, where no significant difference was observed.

**Supplementary Information:**

The online version contains supplementary material available at 10.1186/s13065-024-01117-2.

## Introduction

In recent decades, benign chemistry (green chemistry) has become a preferred method for all chemists, particularly in analytical chemistry, to reduce organic solvent usage and energy consumption. One of the strategies for eco-friendly liquid chromatographic techniques is solvent replacement, which is done here by using ethanol instead of any other organic solvent due to the availability and safety of ethanol [1].

**Fig. 1:**
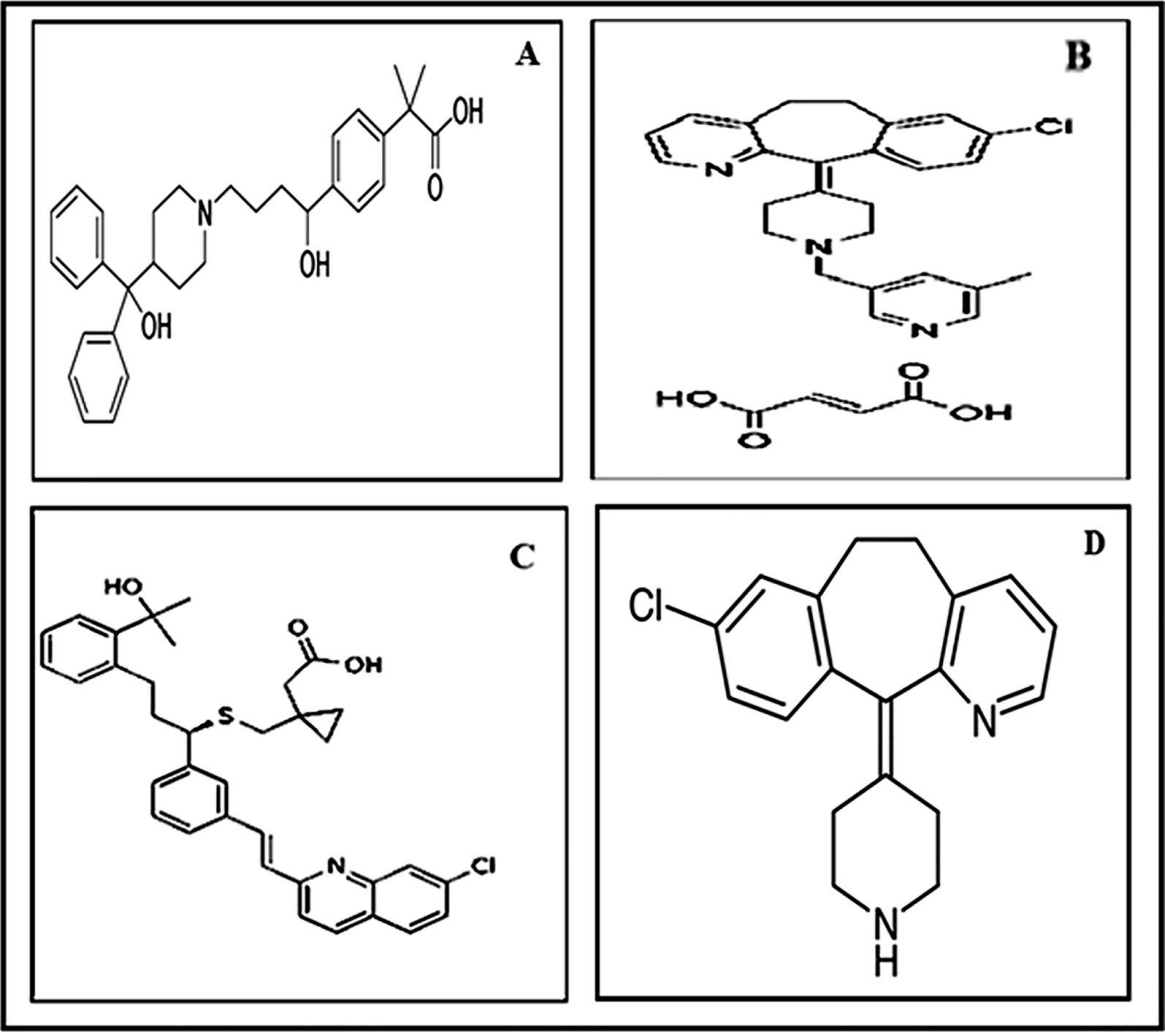
Chemical structures of: **A**: FEX **B**: RUP **C**: MKT **D**: DES

Fexofenadine hydrochloride (FEX); Fig. 1A is 2-[4-[(1RS)-1-Hydroxy-4-[4(hydroxydiphenylmethyl)piperidin-1-yl]butyl]phenyl]-2-methyl propanoic acid hydrochloride [2], is a terfenadine active metabolite. It is a non-sedating antihistaminic medicine of the second generation used in the symptomatic treatment of allergic conditions, including seasonal allergic rhinitis and chronic urticaria, as the hydrochloride salt. In the United Kingdom, a dose of fexofenadine hydrochloride of 120 mg once daily is prescribed to treat seasonal allergic rhinitis; 180 mg once daily is advised for chronic idiopathic urticaria. Additionally, fexofenadine is used in combination with a decongestant, such as pseudoephedrine hydrochloride[3]. FEX can be determined by spectrophotometry [4], RP-HPLC [5], voltammetry [6], and spectrofluorometry [7]. Rupatadine fumarate (RUP); Fig. 1B is 8-Chloro-11-[1-[(5-methylpyridin-3-yl)methyl] [piperidin-4-ylidine]-6,11-dihydro-5H-benzo[5,6]cyclohepta[1,2-b]pyridine(2E)-but-2-enedioate [2]. Rupatadine is a second-generation non-sedating antihistaminic medicine which is selective and long-acting with a strong antagonist activity toward both histamine H1 receptors and platelet-activating factor receptors [8]. RUP can be determined via UV spectrophotometry [9], RP-HPLC [10], voltammetry [11], HPTLC [12], and by non-aqueous titration technique [13]. Montelukast sodium (MKT); Fig. 1C is sodium [1-[[[1R)-1-[3-(E)-2-(7-choroquinolin-2-yl)ethenyl] phenyl]-3-[2-(1-hydroxy)methylethyl)phenyl]propyl]sulfanyl]methyl]cyclopropyl]acetate [2]. MKT is a cysteinyl leukotriene receptor antagonist that has been found to be beneficial in treating allergic rhinitis and asthma. Montelukast is an orally active drug that inhibits physiologic actions of leukotriene receptors at the cysteinyl leukotriene receptor (CysLT1) without any agonist activity [14]. MKT can be determined by UV spectrophotometry [15], RP-HPLC [16], spectrofluorometry [17], and voltammetry [18]. Desloratadine (DES); Fig. 1D is 8-chloro-11-(piperidin-4-ylidine)-6,11-dihydro-5H-benzo[5,6]cyclohepta[1,2-b]pyridine[2]. DES is a novel, non-sedating H1 receptor antagonist, with good tolerance and to be effective in management of seasonal allergic rhinitis, perennial allergic rhinitis and chronic urticaria [19]. DES can be determined via UV spectrophotometry [20], RP-UPLC [21], spectrofluorometry [22] and voltammetry [23].

Rupatadine, desloratadine, fexofenadine, and montelukast are approved medications in British pharmacopeia [2] and United States pharmacopeia [24]. DES and MKT are co-formulated in a tablet dosage form called Telekast D^®^ and Desolid-M^®^ tablets in a pharmaceutical ratio of 1:2 (DES: MKT). This combination is used as a preventive therapy for reducing early and late asthmatic response. It blocks the airway response to inhaled allergen for ≥ 7 h and suppressing eosinophil influx for up to 24 h [25]. DES and MKT were determined simultaneously by RP-HPLC [26–28] and spectrofluorometry [29]. RUP and MKT are co-formulated in a tablet dosage form called Rupanex M^®^ and MontyRup^®^ in a pharmaceutical ratio 1:1. RUP and MKT were determined via RP-HPLC [30, 31], RP-UPLC[32], derivative spectrophotometry [33] and HPTLC [34]. This combination is more beneficial than single one in management of the symptoms of allergic rhinitis and bronchial asthma. A co-formulated tablet using MKT and FEX in a pharmaceutical ratio of 1:12 is known as Afineday^®^, Airway-FX^®^, and Arogora-M^®^ in a pharmaceutical ratio of 1:12 (MKT and FEX). It helps to relieve symptoms of allergy such as sneezing, running nose, watery eyes, itching, swelling, and congestion or stiffness. These compounds were assessed via RP-HPLC [35, 36], HPTLC [37], and UV spectrophotometry [38]. RUP and its potential degradation product, desloratadine, were simultaneously determined using RP-HPLC method [39].

Our study introduces a novel green aqueous RP-HPLC method for determining these antihistaminic drugs in a single run by using an ecofriendly mobile phase of ethanol:water (50:50 v/v, containing 0.04% TEA, and pH 4.5) adjusted by 2 M orthophosphoric acid at 32 °C adjusted by oven and flow rate of 0.85 mL/ min at 220 nm. The run time for separating such combinations is less than 20 min. The studied drugs were evaluated in their pure forms and prescription dosage forms. Some chromatographic responses were optimized by an analytical factorial design as an efficient method for studying multiple factors with a minimal number of trials. Furthermore, decreasing the usage of the organic solvents enhances the positive impact of our method. Because our method is ecofriendly, safer, and requires less energy and time than conventional methods, its greenness can be easily determined.

## Experimental

### Apparatus

Shimadzu LC-20AD Prominence liquid chromatograph (Japan) equipped with a 20 µL Rheodyne injection loop, SPD-20A UV-Vis detector, a column oven (CTO-20A), LC-40D pump, a degasser unit (DGU -207) accomplished by Shimpick Cyano column (250 mm × 4.6 mm i.d., 5-μm particle size). The mobile phase was filtered using 0.45 µm filter membranes in the filter unit.the optimization of the proposed method was manipulated by full factorial design using MINITAB^®^ (release 16 for windows, State College, Pennsylvania, PA, and USA). The greenneess application (AGREE) has been downloaded for free at https://mostwiedzy.pl/AGREE.

### Materials and solvents

The euthentic raw materials are described as followed; Montelukast sodium (99.7 %) was presented by Hikma Pharma Company, Giza, Egypt (batch number:MT17020021), stored in dark glass vials, rupatadine fumarate (99.8 %) was obtained from Mash premiere, 5th settlement, New Cairo, Egypt,fexofenadine (99.68 %) was supplied by El-Obour Modern Pharmaceutical Industries, Cairo, Egypt,and finally desloratadine (99.95 %) was kindly provided by Eva Pharma, Cairo, Egypt, while the pharmaceutical dosage forms are described as followed; Singulair^®^ tablets labeled to contain 10.0 mg MKT, which was produced by Global Nabi Pharmaceuticals, 6th of October, Giza, Egypt,Hisatrup^®^ tablets, each containing 10.0 mg RUP, are produced by Mash premiere, 5th settlement, New Cairo, Egypt, Desa^®^ tablets each containing 5.0 mg of DES, are produced by Delta pharma, 10th of Ramadan City, A.R.E,and finally Fexon^®^ tablets contain 180.00 mg (batch # 139) and are produced by Hikma Pharma Company, Giza district, Egypt. Different solvents were used throughout the method like filtered deionized water and ethanol of HPLC grade, and was obtained from Fisher, UK. The other reagents that also utilized are Orthophosphoric acid (95% purity) was purchased from El- Nasr Company, Cairo, Egypt, HPLC grade ,and Triethylamine (99.9%) was obtained from Sigma Aldrich, HPLC grade.

### Chromatographic conditions

The isocratic technique was used to manipulate an eluent comprising an equal amount of ethanol and water (50:50 v/v) and 0.04% TEA, adjusted by 2 M O-PA to reach pH 4.5 at 32° with UV detection at 220 nm and a flow rate of 0.85 mL/min on a Shimpick Cyano column (250 mm × 4.6 mm i.d., 5-μm particle size). The mobile phase was degassed by sonication after filtration using a 0.45 µm Millipore membrane filter for 10 min.

### Standard solutions

A standard stock solution of 100 µg/mL is prepared by dissolving 10 mg of each drug (RUP, DES, FEX, and MKT) in methanol in 100 mL single volumetric flasks. The working standard solutions were obtained by subsequent dilution of the stock solutions with the mobile phase. All solutions were kept in the refrigerator at 4 °C for three days for MKT and RUP in aluminum foil.

### General procedures

#### Calibration graphs

Aliquots of RUP, DES, FEX, and MKT working standard solutions were transferred to series of 10 mL volumetric flasks for dilution by mobile phase to reach the linear range of 1–10 µg/mL for RUP, DES, and MKT, and 1–24 µg/mL for FEX and mixed well. 20 µL of each concentration was injected triplicate and eluted under the previous optimal chromatographic conditions. The peak areas were plotted against final concentrations of each drug in µg/mL to obtain the corresponding calibration curves then the regression equations were obtained.

#### Assay of the laboratory-prepared mixtures

Different synthetic mixtures of RUP/MKT, DES/MKT, and MKT/FEX were prepared in their pharmaceutical ratio of 1:1, 1:2, and 1:12, respectively. The stock solutions were prepared and then serially diluted to prepare the working standard solutions. Then, the percentage recoveries were then calculated from the corresponding regression equations.

#### Assay of DES as a degradant product of RUP in pure form

DES is the byproduct of RUP [39]. Therefore, a synthetic mixture of DES and RUP in a ratio of 1:10 was prepared using the same procedure described under “Calibration graph development.” The percentage recoveries were then calculated from the corresponding regression equations.

#### Assay of pharmaceutical preparations

##### Single tablets

Ten tablets of Hisatrup (for RUP), singulair tablets (for MKT), DESA tablets (for DES) and fexon tablets (for FEX) were weighed and ground to fine powder individually. The amount of powdered tablets equivalent to 10 mg of each drug is dissolved in 80 mL methanol in 100 mL volumetric flask by sonication and the volume completed to volume with the same solvent and filtered. The procedure under calibration graph was applied. The nominal contents were computed from the regression equation.

##### Co-formulated tablets

Laboratory-prepared tablets of RUP and MKT in their commercial ratio of 1:1 was prepared by mixing 10.0 mg of RUP and 10.0 mg of MKT, and prepared tablets of DES, and MKT in their ratio of 1:2 by mixing 10.0 mg of DES and 20.0 mg of MKT. Finally, prepared tablets of MKT and FEX in their w/w ratio of 1:12 were prepared by mixing 10.0 mg of MKT and 120.0 mg of FEX. All prepared tablets were prepared from powder of single formulation of each drug. After preparing the working standard solutions from the pharmaceutical preparations of each drug as listed in single tablets, appropriate and accurate amounts of them were mixed to achieve the pharmaceutical ratio for each combined tablet; then, the “calibration graph development” procedures were followed. The regression equation was used to calculate each tablet content.

#### Methodology of greenness assessment

The Analytical Greenness (AGREE) is a metric novel green assessment method that depends on the 12 principles of green analytical chemistry (GAC), which are abbreviated as SIGNIFICANCE. AGREE is comprehensive (by incorporation of each of the 12 principles), flexible (by the possibility to assign weights), easy to interpret (the output is a colored pictogram, showing the structure of weak and strong points), easy to perform (with a user-friendly GUI software), fast (The analysis can be performed in a few minutes), and straightforward. It appears to be a clock watch. Every parameter was scored from 0–1. The parameters were numbered from 1 to 12. The colors ranging from light green to darker ones, orange, yellow, and red. When the score is one or nearly one, green shading appears, and when the score is less than one, it changes to yellow or red. The compiled version of the software is downloadable from https://mostwiedzy.pl/AGREE, and the code is available at git.pg.edu.pl/p174235/AGREE.

## Results and discussion

We aim to perform a quantitative assay for RUP, DES, FEX, and MKT in a single run with minimal environmental hazards and be more eco-friendly and usable for convenient routine analysis in quality control than existing methods. Many trials and errors were conducted, starting with the use of the micellar liquid chromatography technique, which resulted in a long retention time. Instead of the toxic acetonitrile or methanol used in most RP-HPLC procedures, a green aqueous RP-HPLC was applied using ethanol as an organic solvent. To be more practical, RP-HPLC technology produces more waste than other existing techniques. However, using ethanol as an organic solvent, water as a buffer, and a somewhat shorter analytical time could result in a positive outcome for the GAC pathway. An optimization approach depending on full factorial design was employed for developing an HPLC- UV method for simultaneaous determination of four antihistaminic drugs (Table 1).

**Table 1:** A 2^3^ full factorial experimental design for RP-HPLC method with UV-detector for separation of DES, RUP, FEX and MKT

Std orders	Run order	Center point	Blocks	pH	TEA	Eth	T_r_	R_s_	T_f_	Std orders	Run order	Center point	Blocks	pH	TEA	Eth	T_r_	R_s_	T_f_
2^3^ full factorial design for FEX										2^3^ full factorial design for MKT									
3	1	1	1	3.50	0.04	45	16.20	2.16	1.46	8	1	1	1	4.50	0.04	50	14.3	3.45	1.21
6	2	1	1	4.50	0.03	50	11.50	2.59	1.10	4	2	1	1	4.50	0.04	45	29.00	3.21	1.40
5	3	1	1	3.50	0.03	50	12.30	2.23	1.40	7	3	1	1	3.50	0.04	50	17.00	1.90	1.23
2	4	1	1	4.50	0.03	45	16.00	1.50	1.33	5	4	1	1	3.50	0.03	50	15.40	2.75	1.35
7	5	1	1	3.50	0.04	50	11.60	2.30	1.82	2	5	1	1	4.50	0.03	45	31.00	3.19	1.41
8	6	1	1	4.50	0.04	50	11.30	3.07	0.93	6	6	1	1	4.50	0.03	50	14.90	2.90	1.62
4	7	1	1	4.50	0.04	45	19.50	1.62	1.64	1	7	1	1	3.50	0.03	45	29.00	2.70	1.42
1	8	1	1	3.50	0.03	45	21.00	2.40	1.53	3	8	1	1	3.50	0.03	45	28.00	2.80	1.30
2^3^ full factorial design for DES										2^3^ full factorial design for RUP									
4	1	1	1	4.50	0.04	45	11.00	2.60	1.52	5	1	1	1	3.50	0.03	50	11.14	2.37	1.16
1	2	1	1	3.50	0.03	45	10.54	0.80	1.84	2	2	1	1	4.50	0.03	45	13.00	2.91	1.44
3	3	1	1	3.50	0.04	45	10.43	0.82	1.83	4	3	1	1	4.50	0.04	45	12.30	3.33	1.29
8	4	1	1	4.50	0.04	50	8.00	3.75	1.42	3	4	1	1	3.50	0.04	45	13.10	2.81	1.44
5	5	1	1	3.50	0.03	50	8.60	1.75	1.60	8	5	1	1	4.50	0.04	50	9.72	3.21	1.11
2	6	1	1	4.50	0.03	45	10.0	1.96	1.68	7	6	1	1	3.50	0.04	50	10.50	2.81	1.10
6	7	1	1	4.50	0.03	50	8.20	2.91	1.45	1	7	1	1	3.50	0.03	45	13.000	2.51	1.54
7	8	1	1	3.50	0.04	50	8.50	2.60	1.40	6	8	1	1	4.50	0.03	50	9.64	2.78	1.26

### Method development and validation

The optimum result of coupling GAC techniques and studying variable parameters is listed in Table 1 to ensure the best separation, resolution, and sensitivity.

#### Choice of the analytical column

Four analytical columns were studied to find the best one in separation, resolution, and optimum retention time; the first column is thermo scientific C18 Hypersil column (250 mm × 4.6 mm i.d., 5-μm particle size), USA, then thermo scientific C8 Hypersil column (250 mm × 4.6 mm i.d., 5-μm particle size), USA, in addition to the thermo scientific phenyl Hypersil column (250 mm × 4.6 mm i.d., 5-μm particle size), USA, and finally Shimpick cyano column (250 mm × 4.6 mm i.d., 5-μm particle size).

The best column was the Shimpick cyano column because it resulted in the best separation, resolution, and retention time, whereas others caused peak overlapping (Thermo Scientific phenyl Hypersil column), peak broadening (Thermo Scientific C8 Hypersil column), peak splitting, or higher retention time (Thermo Scientific C18 Hypersil column).

#### Choice of best wavelength

**Fig. 2:**
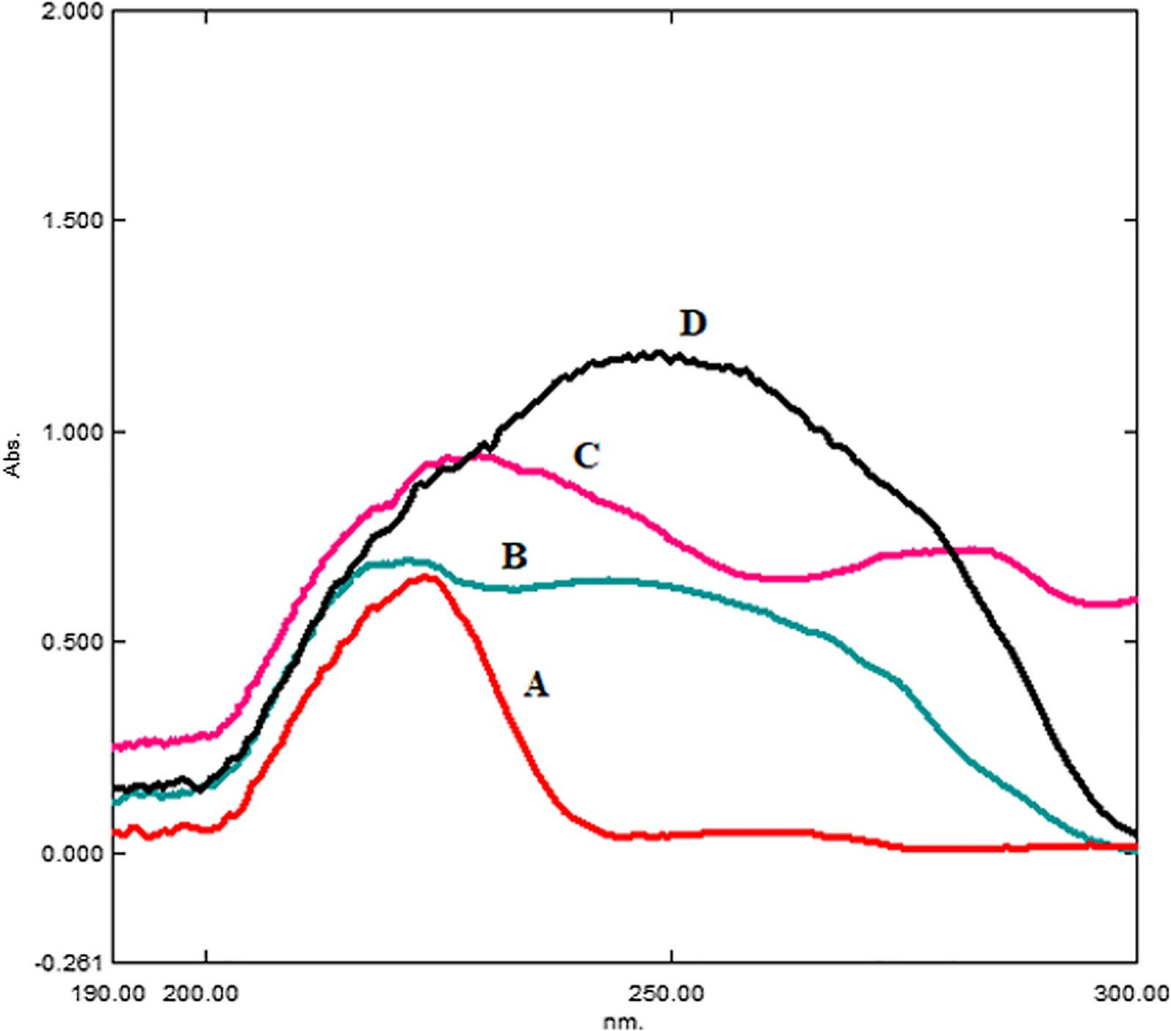
UV Spectra of ethanolic solutions of the studied drugs:** A**: FEX **B**: RUP **C**: MKT **D**: DES

The wavelength has a significant impact on the sensitivity of our method. After scanning different wavelengths such as 210, 220, 250, and 280 nm and intercepting λ maxima (λ _max_) for all drugs, we found that 220 nm is optimal for their sensitivity and selectivity. Although 210 nm has higher sensitivity and better intensity, it also has additional noise due to the ethanol cut-off. Figure 2 shows the different UV spectra of all drugs.

#### Mobile phase composition

Because ethanol is one of the least hazardous organic solvents and has a lower vapour pressure, which causes less evaporation and, hence, less amounts to be inhaled, it is the best option for our technique, which is based on an environmentally friendly approach for analyzing these antihistaminic combinations. Additionally, ethanol is more widely accessible and less expensive than other common organic solvents [40].

The proposed technique employs factorial design to obtain optimum parameters for separation as it saves time and resources. The studied drugs were resolved using isotonic elution of aqueous mobile phase consisting of ethanol, small amount of triethylamine in less than 17 min. The mobile phase pH was found to greatly effect the retention time of (tR) of the studied drugs. Different pH values (2.5–7) were tested to study their effect on the resolution of peaks. The separation occurred at pH from 3.5 to 4.5 as lower pH than 3.5 results in overlapping od RUP and DES. However, great overlapping and fragmentation of peaks was observed if pH ≥ 5.

The ratio of ethanol to water was studied from 45 to 65%. When the amount of ethanol is less than 47, a significant broadening and delay in the retention time are observed. Increasing the percentage above 53% resulted in overlapped peaks with no base-to-base separation.

The presence of triethanolamine (TEA) is crucial in this study because it enhances the peak form by decreasing tailing. The percentage of TEA that is studied ranges from 0.03 to 0.07%. In absence of TEA, peak broadening and forking of peaks occurred, whereas increasing its percentage causes short analysis time but more overlapping between peaks and hinders baseline separation.

Although ethanol serves as the foundation for this study, it has two major limitations. The first limitation is the appearance of its cut-off at 210 nm, which we avoided by using 220 nm. The second limitation is that the viscosity of EtOH/water mixtures causes high backpressures, thus limiting the use of EtOH with conventional LC systems [41]. This was solved by raising the temperature to 32°, after which overlapped peaks were detected.

After the examination of different flow rates from 0.8 to 1.2 mL / min, it was found that the optimal one with a high NTP is 0.85 mL/min. Flow rates > 0.85 mL/min resulted in low resolution, whereas those < 0.85 mL/min resulted in unacceptable retention times.

Three independent factors were studied using experimental design to choose the most optimum conditions for resolving each overlapping peaks of the studied drugs in mixtures. These factors include different pH values from 3.5 to 4.5 as well as different ratios of ethanol from 45 to 50% and triethylamine from 0.03 to 0.04%.

### Experimental design

MINITAB^®^ employs a 2^3^ full factorial analytical design as a type of design of experiment applied by MINITAB^®^. This multivariate system provides all the simultaneous factors in one, as opposed to the normal optimization technique, which changes one factor while fixing the other factors, consuming time, money, and solvents, all of which harm the environment. The m ^k^ or 2^3^ full factorial design is characterized by having three independent factors with two levels each [42]. It enables the statistical studying of multiple independent and dependent variables at the same time using the two minimum and maximum levels (−1,1) entered into the design. These levels are defined by carrying out some initial chromatographic experiments before conducting an experimental design. It enables the studying of 2–15 factors through one design using minimal trials, hence decreasing the consumption of the mobile phase. The independent variables are the pH, ethanol, and TEA percentages, while the optimized dependent variables are the retention time, resolution, and tailing factor for each drug. By entering the two levels for each factor, the full factorial design creates eight trials in which the optimum mobile phase will be included. The first factor is pH (A), where the suitable values used for separation are between 3.5 and 4.5. The second factor is the ethanol ratio (C), which works between 45 and 50, and the final one is the TEA percentage (B), which is between 0.03 and 0.04 [43]. These values were inputted after a series of trials and errors to determine the best retention time, resolution, and tailing factor. This design was done for RUP, DES, FEX, and MKT, illustrated in Tables 1 and 2. Eight experiments were supposed to be from this 2^3^ factorial design, explaining the interaction between the responses and the different levels of the independent variables like pH, percentage of triethanolamine, and the ratio of ethanol. This method could also be used to validate other methods, such as the robustness of the method.

**Table 2:** Response optimization of 2^3^ factorial design for RP-HPLC–UV separation of DES, RUP, FEX and MKT^*^

Response	Goal	Lower	Target	Upper	Weight	Importance	Predicted responses	Desirability	Composite desirability
Parameters									
MKT									
T_r_	Target	13	14.3	15	1	1	14.30	1	0.779
R_s_	Target	3	3.45	4.5	1	1	3.45	1	
T_f_	Target	0.9	1.11	1.3	1	1	1.21	0.474	
FEX									
T_r_	Target	10	11.3	12	1	1	11.30	1	1.00
R_s_	Target	0.9	0.93	1.2	1	1	0.93	1	
T_f_	Target	3	3.07	4	1	1	3.07	1	
DES									
T_r_	Target	7	8	9	1	1	8	1	1.00
R_s_	Target	2.6	3.75	4.5	1	1	3.75	1	
T_f_	Target	1.4	1.4	1.68	1	1	1.42	1	
RUP									
T_r_	Target	8	9.72	10	1	1	9.72	1	1.00
R_s_	Target	3	3.21	4.5	1	1	3.21	1	
T_f_	Target	0.9	1.11	1.5	1	1	1.11	1	

^*^The optimum conditions: Ethanol:Water (50:50), pH = 4.5, %TEA = 0.04, Flow rate = 0.85 mL/min, Temp. = 32 °C

The optimized conditions were determined by the optimizer response minitab^®^ via calculating desirability ranges from (0–1); zero means there is no matching between the independent variables and their levels and the responses, so there are no optimum parameters, while one or nearer to one means acceptable or optimum conditions were discovered and could be applied through the method. Factorial design can be used for optimization of an analytical method or the robustness of the method. In this study, we used a full factorial design for both the method optimization (design 2^3^) and the robustness (design 2^3^).

**Fig. 3:**
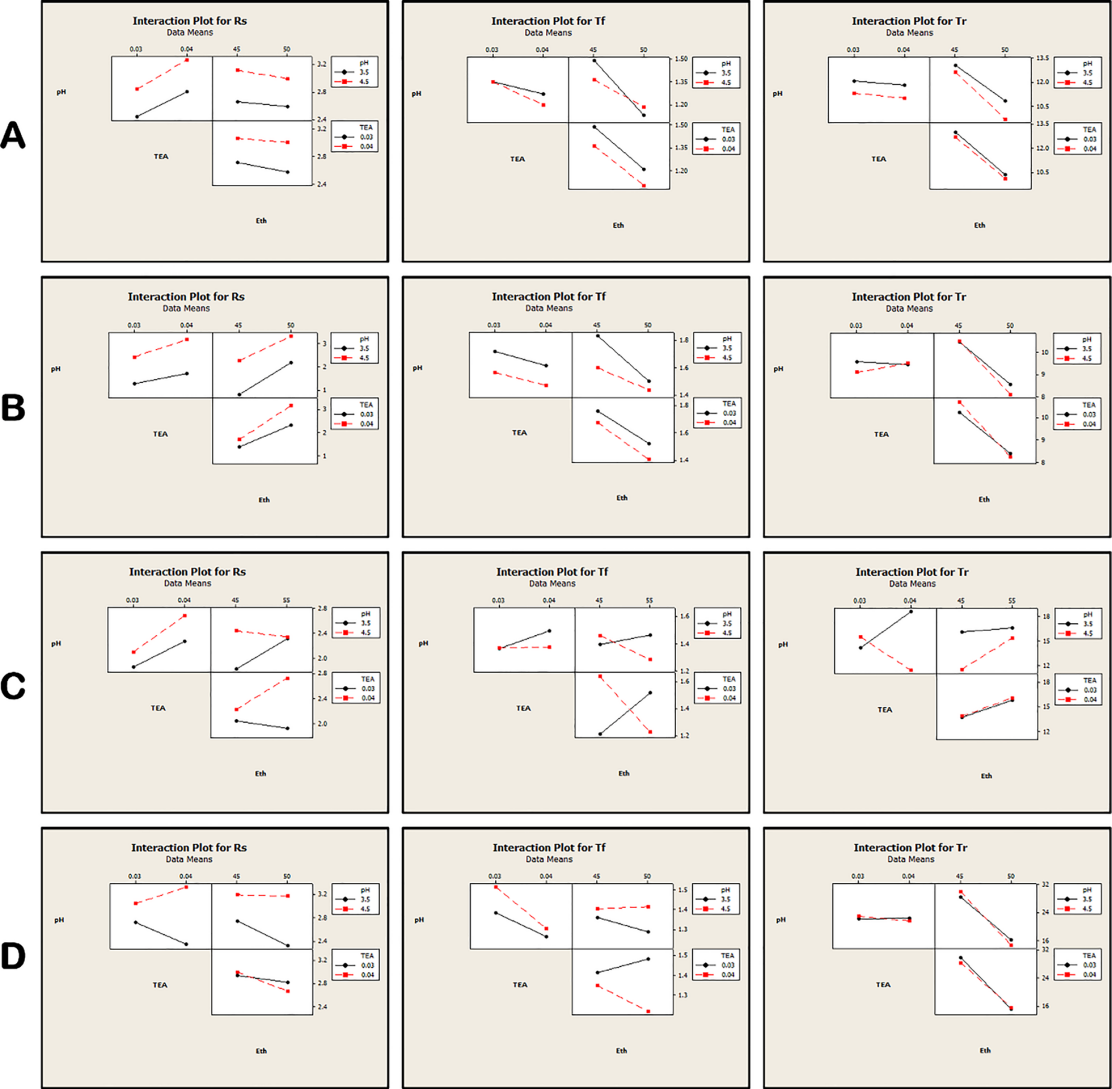
The interaction plots of the chromatographic responses at α = 0.05 where **A**: RUP **B**: DES **C**: FEX **D**: MKT

According to the Pareto charts in Fig. 3; the percentage of ethanol has a significant effect on the retention time for RUP, DES and MKT while the tailing factor is not affected by changing the different selected parameters except for RUP that is affected by increasing the percentage of ethanol. The resolution of RUP and DES are deeply affected by pH, TEA and the ethanol percentage while the resolution of FEX and MKT are not affected.

Factorial design yields main plots and interaction plots for determining whether these individual parameters had an effect on the response. For desloratadine, rupatadine, fexofenadine, and montelukast, as the TEA, ethanol, and pH increased in the specified ratio; a decrease in the retention time (tr), increase resolution (rs), and decrease in tailing (tf), were reflected in our results.

The polynomial equation for this experimental design:

$${\text{R }} = \, \beta_{0} + \, \beta_{{1}} {\text{A }} + \, \beta_{{2}} {\text{B }} + \, \beta_{{3}} {\text{C }} + \, \beta_{{2}} {\text{AB }} + \beta_{{2}} {\text{BC }} + \, \beta_{{2}} {\text{AC }} + \, \beta_{{2}} {\text{A}}^{{2}} + \, \beta_{{2}} {\text{B}}^{{2}} + \, \beta_{{2}} {\text{C}}^{{2}}$$

where R denotes the response, β denotes the regression coefficients, and A, B, and C denote the pH, TEA, and ethanol ratio, respectively.

The significance of the independent variables on the responses and interaction between them was estimated by the Fisher statistical test for variance analysis (ANOVA) model [44].

Finally, the proposed mobile phase composed of 50:50 ethanol and water containing 0.04% triethylamine at pH 4.5 adjusted by ortho phosphoric acid (O-PA) and flow rate 0.85 mL / min at a temperature 32^◦^ C.

## Method validation

This separation study was validated in accordance with the Q2R1 Guidelines of the International Council of Harmonization [45]. The metrics analyzed and addressed in Table 3 include linearity, accuracy, precision, LOD, LOQ, robustness, and system suitability.

**Table 3:** Analytical performance data for determination of the RUP, DES, FEX and MKT by the proposed method

Parameter	RUP	DES	FEX	MKT
Range (µg/mL)	1–10	1–10	1–24	1–10
Intercept (a)	69.257 × 10^3^	28.005 × 10^3^	35.029 × 10^3^	7.101 × 10^3^
Slope (b)	67.954 × 10^3^	39.874 × 10^3^	62.346 × 10^3^	68.516 × 10^3^
Correlation coefficient (*r*)	0.9998	0.9997	0.9999	0.9999
S.D. of residuals (S_y/x_)	6.420 × 10^3^	4.369 × 10^3^	8.501 × 10^3^	3.841 × 10^3^
S.D. of intercept (S_a_)	5.342 × 10^3^	3.636 × 10^3^	5.628 × 10^3^	3.196 × 10^3^
S.D. of slope (S_b_)	0.834 × 10^3^	0.568 × 10^3^	0.461 × 10^3^	0.499 × 10^3^
Percentage relative standard deviation (% RSD)	1.05	1.22	1.36	0.99
Percentage relative error (% Error)	0.472	0.544	0.60	0.44
Limit of detection, LOD (µg/mL)	0.26	0.30	0.27	0.15
Limit of quantitation, LOQ (µg/mL)	0.78	0.91	0.82	0.47

### Linearity and concentration range

Under optimum analytical conditions, a linear relationship was established between the drug concentration and the peak area in the range of 1–10 µg/mL for RUP, DES, and MKT and 1–24 µg/mL in the case of FEX. Table 3 shows the regression parameters produced following statistical analysis, which included a higher correlation coefficient and a lower standard deviation [46].

The following equations were derived from the regression data analysis:

$${\text{P }} = {67}.{954} \times {1}0^{{3}} {\text{C }} + { 69}.{257} \times {1}0^{3} \quad (r = 0.{9998}){\text{ For RUP}}$$

$${\text{P }} = {39}.{874} \times {1}0^{{3}} {\text{C }} + {28}.00{5} \times {1}0^{3} \quad \left( {r = \, 0.{9997}} \right){\text{ For DES}}$$

$${\text{P }} = {62}.{346} \times {1}0^{{3}} {\text{C }} + {35}.0{29} \times {1}0^{3} \quad \left( {r = 0.{9999}} \right){\text{ For FEX}}$$

$${\text{P }} = {68}.{516} \times {1}0^{{3}} {\text{C }} + {7}.{1}0{1} \times {1}0^{3} \quad \left( {r = \, 0.{9999}} \right){\text{ For MKT}}$$

where: P is the peak area, *r* is the correlation coefficient, and C is the concentration in µg/mL.

### Limit of detection and quantitation

As shown in Table 3, LOD is the lowest concentration that could be detected and calculated at 3.3 S_a_/b, while LOQ is the lowest concentration that could be quantified in terms of accuracy and precision and calculated at 10 S_a_/b.; where S_a_ means that the standard deviation of the intercept of the regression line and b is the slope of the calibration graph.

### Accuracy

Different concentrations (1–10 µg/mL) covering the calibration range for RUP, DES, and MKT, and from 1–24 µg/mL for FEX reveal the accuracy. In comparison to previously published approaches, the student t-test and variance ratio F-test [46] were applied for statistical analysis by comparison of the results of the proposed method with these obtained from comparison methods of the studied drugs [28, 31, 36]. There was no significant difference between the performance of the suggested and the comparison methods in terms of accuracy and precision (Table 4).

**Table 4:** Assay results for determination of the RUP, DES, FEX and MKT in pure forms

Analyte	Proposed method			Comparison methods [28, 31, 36]		Analyte	Proposed method			Comparison methods [28, 31, 36]	
	Concn. Taken (μg/mL)	Concn. Found (μg/mL)	% Found^a^	Concn. Taken (μg/mL)	% Found^a^		Concn. Taken (μg/mL)	Concn. Found (μg/mL)	% Found^a^	Concn. Taken (μg/mL)	% Found^a^
RUP	1.00	1.005	100.50	20.00	98.65	FEX	1.00	0.986	98.60	10.00	101.41
	2.00	2.025	101.25	30.00	101.74		2.00	1.974	98.70	20.00	98.72
	6.00	5.965	99.42	40.00	99.36		8.00	7.904	98.80	30.00	100.42
	8.00	7.901	98.76				10.00	10.180	101.80		
	10.00	10.096	100.96				24.00	23.960	99.83		
Mean ± SD			100.18 ± 1.05		99.92 ± 1.62	Mean ± SD			99.55 ± 1.35		100.18 ± 1.36
*t*			0.28 (2.45)^b^			*t*			0.64 (2.45)^b^		
*F*			2.35 (6.94)^b^			*F*			1.01 (6.94)^b^		
DES	1.00	1.003	100.30	40.00	99.44	MKT	1.00	1.014	101.40	40.00	99.68
	2.00	2.009	100.45	50.00	100.87		2.00	1.986	99.30	50.00	100.54
	6.00	5.904	98.40	60.00	99.65		6.00	5.957	99.28	60.00	99.77
	8.00	8.134	101.68				8.00	8.076	100.95		
	10.00	9.948	99.48				10.00	9.966	99.66		
Mean ± SD			100.06 ± 1.22		99.99 ± 0.77	Mean ± SD			100.12 ± 0.99		100.00 ± 0.47
*t*			0.09 (2.45)^b^			*t*			0.19 (2.45)^b^		
*F*			2.48 (19.25)^b^			*F*			4.38 (19.25)^b^		

^a^Each result was the average of three separate determinations

^b^The tabulated* t* and *F* values at P = 0.05 [46]

The comparison method used for determination of RUP and MKT was achieved through the chromatographic separation on hibar R 250-4, C-18 columns (250 mm × 4.6 mm,5um) using a mobile phase consisting of Methanol: Water (90:10v/v) with 0.1% Triethyl amine pH 3.41 adjusted with ortho phosphoric acid at a flow rate of 1 ml/min. Detection wavelength was found 260 nm [28].

The comparison method used for determination of FEX and MKT was achieved through the chromatographic separation on a Phenomenex C column (150 × 4.6 mm i.d, particle size of 5µ) using a mixture of 0.1 M potassium dihydrogen 18 orthophosphate buffer (pH 5.0) and methanol in the ratio of 60:40 v/v as mobile phase in an isocratic elution mode, at a flow rate of 1 ml/min. The detection was monitored at 220 nm [31].

The comparison method used for determination of DES and MKT was achieved through the chromatographic separation on a reversed-phase C-18 column (250 mm × 4.8 mm i.e., particle size 5 µm) column with mobile phase consisting of methanol: water: Acetic acid (90:10:0.05 v/v/v) was used. The flow rate was 1.0 ml/ min and effluents were monitored at 280 nm [36].

The proposed method utilized ethanol and water as green solvents compared to other methods that used methanol or other additions which are less green than our method. Also, the proposed method could efficiently separate the four antihistaminic drugs in a single shot that is firstly introduced in the proposed study.

### Precision

Precision was evaluated in Table 5 by intra-day precision through injecting three concentrations of each drug triplicate on the same day and interday precision through injecting three concentrations of each drug in three successive days.

**Table 5:** Precision data for determination of RUP, DES, FEX and MKT by the designed method

Concentration (μg/mL)		RUP			DES				FEX			MKT		
		1.00	8.00	10.00	1.00	8.00	10.00		1.00	8.00	10.00	1.00	8.00	10.00
Intra-day	%Recovery (mean)^a^	99.60	99.41	100.34	100.30	100.62	100.01		100.56	100.25	98.97	100.13	100.25	98.24
	± SD	0.90	0.58	0.36	0.70	0.99	0.52		1.56	0.63	0.63	1.70	0.63	0.19
	%RSD	0.90	0.60	0.40	0.69	0.99	0.52		1.55	0.63	0.64	1.69	0.63	0.19
	%Error	0.52	0.33	0.21	0.40	0.57	0.30		0.89	0.36	0.36	0.98	0.36	0.11
Inter-day	%Recovery (mean)^a^	99.66	99.88	100.13	99.60	100.56	99.87		99.53	99.60	99.17	100.36	99.70	100.62
	± SD	0.91	1.12	1.25	0.70	1.02	0.46		1.13	1.25	0.46	1.31	1.25	0.89
	%RSD	0.91	1.12	1.25	0.70	1.01	0.46		1.14	1.26	0.47	1.30	1.25	0.89
	%Error	0.53	0.65	0.72	0.41	0.58	0.27		0.66	0.73	0.27	0.75	0.72	0.51

^a^Each result is the average of three separate determinations

### Selectivity

The separation of the four studied drugs without any interference from the additives from the tablet components is a positive sign of selectivity. By testing for excipient interference in the pharmaceutical formulations using this method, the selectivity was evaluated. Talc, magnesium stearate, or lactose did not cause any interference. The obtained % recoveries of the two drugs in their pharmaceutical preparations are from (98.5–102) with RSD (< 2%) for RUP,DES,FEX and MKT, indicating the selectivity of the results.

### Robustness

**Fig. 4:**
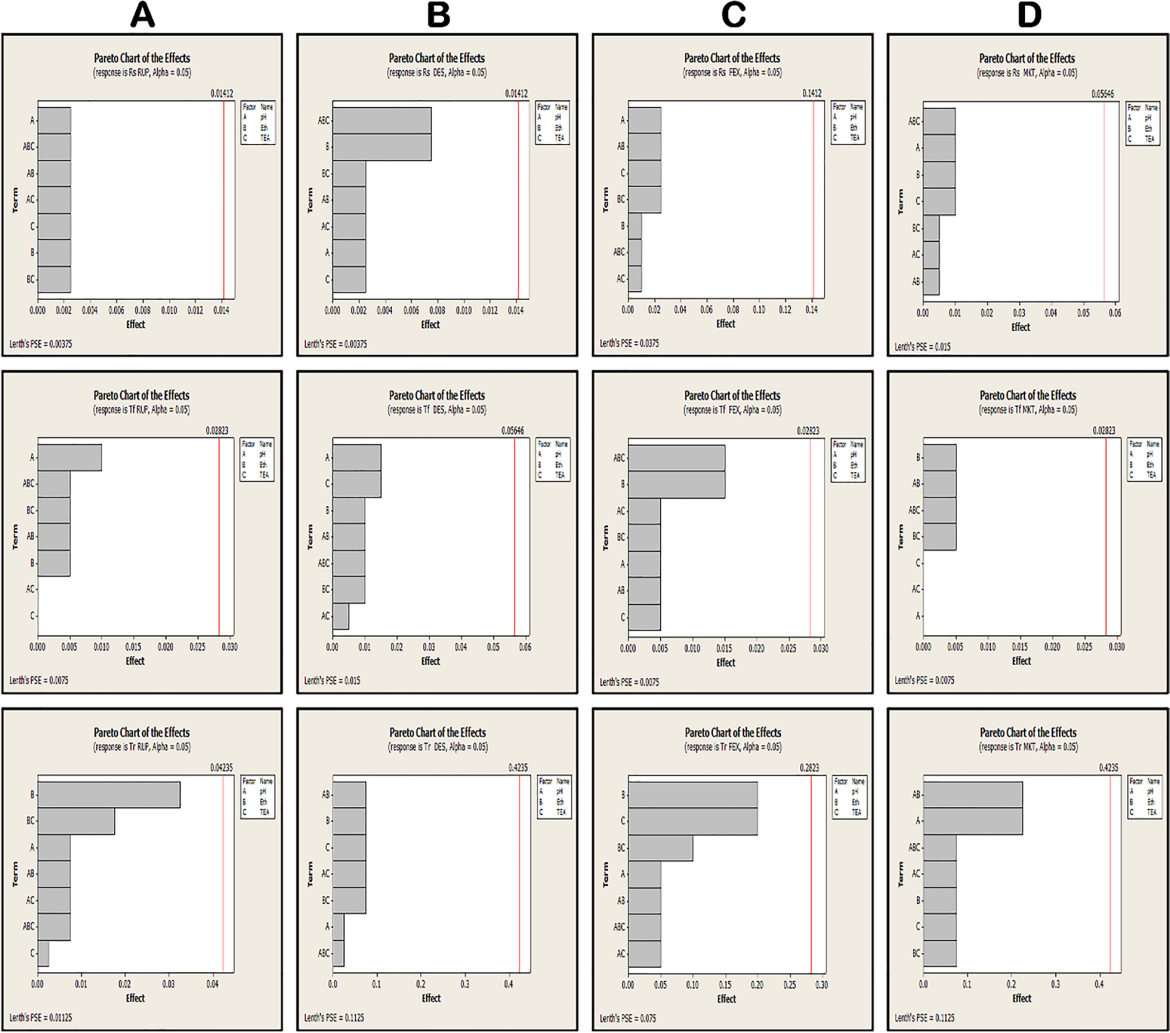
A pareto charts for robustness of the independent variables: (Rs), (T_r_) and (T_f_); **A**: RUP **B**: DES **C**: FEX **D**: MKT

Proving the robustness of the method by making small but deliberate changes in the chromatographic conditions. That did not cause significant changes in the responses. Robustness was illustrated by 2^3^ full factorial design with one center point. This design consists of three factors: pH (A), percentage of triethanolamine (B) and ethanol ratio (C) and each factor has two levels lower (−1) and upper (+ 1) illustrated by the pareto charts in Fig. 4 and Table 6. These independent factors are pH 4.5 (± 0.1), ethanol percentage 50 (± 1%) and TEA 0.04 (± 0.01) which did not affect the peak area of the drugs.

**Table 6:** A 2^3^ full factorial design for robustness of the method

Response	Goal	Lower	Target	Upper	Weight	Importance
T_r_ DES	Target	7.9	8	8.1	1	1
T_r_ RUP	Target	9.7	9.72	9.75	1	1
T_r_ FEX	Target	11.1	11.6	11.7	1	1
T_r_ MKT	Target	14	14.3	14.6	1	1
R_s_ DES	Target	3.74	3.75	3.76	1	1
R_s_ RUP	Target	3.20	3.21	3.23	1	1
R_s_ FEX	Target	3	3.07	3.1	1	1
R_s_ MKT	Target	3.44	3.45	3.46	1	1
T_f_ DES	Target	1.41	1.42	1.46	1	1
T_f_ RUP	Target	1.2	1.21	1.22	1	1
T_f_ FEX	Target	0.9	0.93	0.95	1	1
T_f_ MKT	Target	1.2	1.21	1.22	1	1

## Applications

**Fig. 5:**
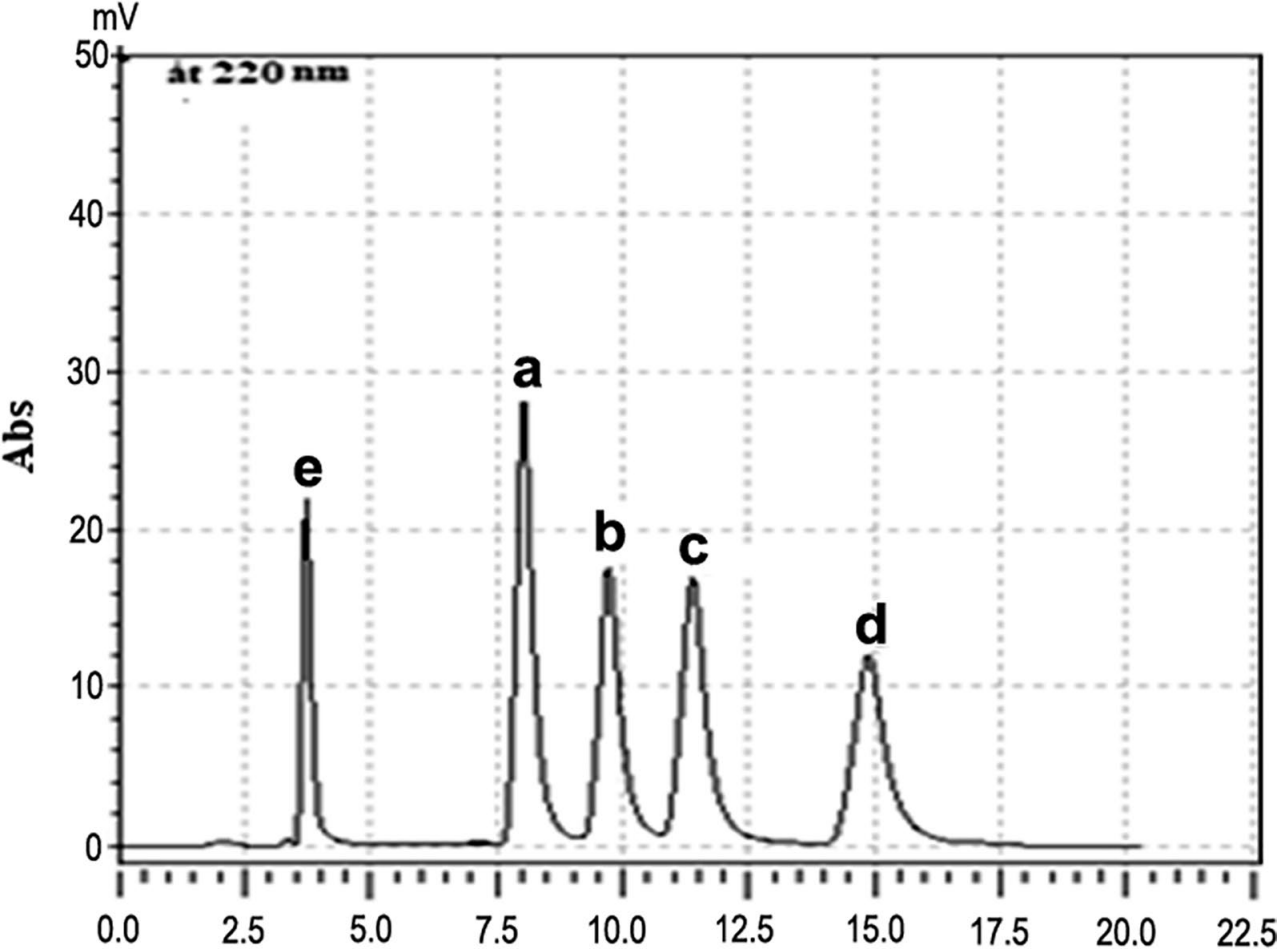
Chromatogram of separation of the four drugs at 220 nm under the optimum chromatographic conditions: 10 µg/mL of **a** DES, **b** RUP, **c** FEX, **d** MKT, while **e** fumarate peak

The suggested HPLC method was applied to the analysis of RUP, DES, FEX and MKT Fig. 5, the laboratory prepared synthetic mixtures of RUP/MKT, DES/MKT and FEX/MKT.

(Table 7) and (Additional file 1: Fig. S1), the studied drugs in their single tablets (Additional file 1: Table S1) and the laboratory prepared combined dosage forms (tablets) (Additional file 1: Table S2). The prosed method could determine desloratadine as a degradation product of rupatadine in a ratio 10:1 (Additional file 1: Fig. S2). In terms of accuracy and precision, these results were in good agreement with those obtained using the comparison methods [28, 31, 36] employing statistical analysis using t and F-test [46].

**Table 7:** Assay results for determination of RUP, DES, FEX and MKT in synthetic mixtures by the proposed method

Analyte	Proposed method						Comparison methods [28, 31, 36]	
	Concn. Taken (μg/mL)		Concn. Found (μg/mL)		% Found^a^		% Found^a^	
Synthetic mixture of RUP/MKT (1:1)	RUP	MKT	RUP	MKT	RUP	MKT	RUP	MKT
	1.00	1.00	1.001	1.001	100.10	100.10	99.12	99.68
	8.00	8.00	7.901	8.008	98.76	100.10	101.13	100.54
	10.00	10.00	10.009	9.970	100.09	99.70	99.58	99.77
Mean ± SD					99.65 ± 0.77	99.97 ± 0.23	99.94 ± 1.05	100.00 ± 0.47
% Error					0.45	0.13	0.61	0.27
*t*					0.39 (2.78)^b^	0.098 (2.78)^b^		
*F*					1.87 (19)^b^	4.19 (19)^b^		
Synthetic mixture of DES/MKT (1:2)	DES	MKT	DES	MKT	DES	MKT	DES	MKT
	1.00	2.00	0.988	1.982	98.80	99.10	99.44	99.68
	2.00	4.00	2.014	4.027	100.70	100.68	100.87	100.54
	4.00	8.00	3.996	7.991	99.90	99.89	99.65	99.77
Mean ± SD					99.80 ± 0.95	99.89 ± 0.79	99.99 ± 0.77	100.00 ± 0.47
% Error					0.55	0.46	0.45	0.27
*t*					0.26 (2.78)^b^	0.20 (2.78)^b^		
*F*					1.53 (19)^b^	2.79 (19)^b^		
Synthetic mixture of MKT/FEX (1:12)	MKT	FEX	MKT	FEX	MKT	FEX	MKT	FEX
	1.00	12.00	0.991	11.825	99.10	98.54	99.68	101.41
	1.50	18.00	1.519	18.14	101.27	100.78	100.54	98.72
	2.00	24.00	1.99	23.946	99.50	99.78	99.77	100.42
Mean ± SD					99.96 ± 1.15	99.70 ± 1.12	100.00 ± 0.47	100.18 ± 1.36
% Error					0.67	0.65	0.27	0.78
*t*					0.06 (2.78)^b^	0.47 (2.78)^b^		
*F*					5.97 (19)^b^	1.47 (19)^b^		

^a^Each result was the average of three separate determinations

^b^The tabulated* t* and *F* values at P = 0.05[46]

## Greenness assessment

Assessing the greenness of the analytical method has a good impact on the environment. Scientific research will never be stopped until the analytical processes are completely greened, even if it means recycling the waste or synthesizing new safe chemicals for humans and the environment, so the researchers will take major steps toward this goal. Several green metrics have been developed, beginning with the National Environmental Method Index (NEMI) [47], which is based on a four-part pictogram that classifies waste or reagents as bioaccumulative, persistent, toxic, or corrosive. Another metric system that has been developed is the analytical eco scale [48], which is based on calculating the penalty point score. The green analytical procedure index (GAPI) [49] is a novel metric that takes into account all the parameters included in the method development but does not include the number of samples analyzed in one hour. Using the analytical greenness calculator and the AGREE [50] metric, this was overcorrected. The AGREE metric is a novel assessment method that depends on the 12 principles of green analytical chemistry (GAC), which are abbreviated as SIGNIFICANCE. It appears to be a clock watch. Every parameter was scored from 0 to 1, with the ultimate score added in the middle, and in the terminal, the parameters were numbered from 1 to 12. This model is green, with colors ranging from light green to darker ones, orange, yellow, and red. When the score is one or nearly one, green shading appears, and when the score is less than one, it changes to yellow or red. Additional file 1: Table S3 shows the Assessment of greenness of the proposed HPLC method using AGREE metric; there is one red zone due to LC’s high energy consumption of 1.5 Kwh and two yellow zones due to the composition of reagents and the waste disposal. This application can be downloaded for free at https://mostwiedzy.pl/AGREE.

## Conclusion

In our analytical study, we discovered that the replacement of toxic and conventional solvents like methanol and acetonitrile with eco-friendly ones like ethanol and water is a target for GAC because this system is advantageous in the chromatographic separation and has great eluting strength. The green property was conducted using the AGREE metric. Aside from the benign impact of our methodology, it is a sensitive, simple, selective, and easily applicable HPLC method for regular analysis in quality control units. For the first time, the proposed method was developed and validated in bulk, synthetic combination, and dosage forms for the quaternary RUP, DES, FEX, and MKT separation.

### Supplementary Information

**Additional file 1: Table S1.** Determination of RUP, DES, FEX and MKT in pharmaceutical preparations using the proposed method. **Table S2.** Assay results for determination of RUP, DES, FEX and MKT in laboratory prepared tablets by the proposed method. **Table S3.** Assessment of greenness of the proposed HPLC method using AGREE metric. **Figure S1.** Synthetic mixture of: A: 4 µg/mL of DES and 8 µg/mL of MKT. B: 8 µg/mL of RUP and 8 µg/mL MKT. C: 12 µg/mL of FEX and 1 µg/mL of MKT. **Figure S2.** Synthetic mixture of desloratadine (1 µg/mL) as a degradation product of rupatadine (10 µg/mL).

## Data Availability

All data generated or analyzed during this study are included in this published article [and its supplementary information files].
