# Supplementary material for: Environmentally benign liquid chromatographic method for concurrent estimation of four antihistaminic drugs applying factorial design approach
Source: BMC Chem. 2024 Jan 30;18(1):26. doi: 10.1186/s13065-024-01117-2 (PMC10829218; doi:10.1186/s13065-024-01117-2)
Supplement: Supplementary file 1 — Additional file 1: Table S1. Determination of RUP, DES, FEX and MKT in pharmaceutical preparations using the proposed method. Table S2. Assay results for determination of RUP, DES, FEX and MKT in laboratory prepared tablets by the proposed method. Table S3. Assessment of greenness of the proposed HPLC method using AGREE metric. Figure S1. Synthetic mixture of: A: 4 µg/mL of DES and 8 µg/mL of MKT. B: 8 µg/mL of RUP and 8 µg/mL MKT. C: 12 µg/mL of FEX and 1 µg/mL of MKT. Figure S2. Synthetic mixture of desloratadine (1 µg/mL) as a degradation product of rupatadine (10 µg/mL). [file 13065_2024_1117_MOESM1_ESM.docx]

**Environmentally benign liquid chromatographic method for concurrent estimation of four antihistaminic drugs applying factorial design approach**

**Rana Ghonim^a,b^, Manar M. Tolba^a^, Fawzia Ibrahim^a^, Mohamed I. El-Awady^a, b*^**

1. *Department of Pharmaceutical Analytical Chemistry, Faculty of Pharmacy, Mansoura University, Mansoura 35516, Egypt.*
2. *Department of Pharmaceutical Chemistry, Faculty of Pharmacy, Delta University for Science and Technology, International Coastal Road, Gamasa 11152, Egypt.*

**Supporting information:**

**Table S1:** Determination of RUP, DES, FEX and MKT in pharmaceutical preparations using the proposed method.

**Table S2:** Assay results for determination of RUP, DES, FEX and MKT in laboratory prepared tablets by the proposed method.

**Table S3:** Assessment of greenness of the proposed HPLC method using AGREE metric.

**Fig. S1:**  Synthetic mixture of:

A: 4 µg/mL of DES and 8 µg/mL of MKT

B: 8 µg/mL of RUP and 8 µg/mL MKT

C: 12 µg/mL of FEX and 1 µg/mL of MKT

**Fig. S2:** Synthetic mixture of desloratadine (1 µg/mL) as a degradation product of rupatadine (10 µg/mL)

**Table S1:** Determination of RUP, DES, FEX and MKT in pharmaceutical preparations using the proposed method

| Compound | Proposed method | | | Comparison methods ^(28,31,36)^ | |
| --- | --- | --- | --- | --- | --- |
|  | Amount taken (μg/mL) | Amount found  (μg/mL) | % Found | Amount taken (μg/mL) | % Found |
| Hisatrup^®^ tablets  RUP (10.0 mg) | 1.00 | 1.011 | 101.10 | 20.00 | 101.06 |
|  | 8.00 | 7.980 | 99.75 | 30.00 | 98.52 |
|  | 10.00 | 10.001 | 100.01 | 40.00 | 100.57 |
| ‾x ± SD |  |  | 100.28 ± 0.36 |  | 100.05 ± 1.30 |
| *t* |  |  | 0.11 |  |  |
| *F* |  |  | 14.39 |  |  |
| Desa ^®^ tablets  DES (10.0 mg) | 1.00 | 0.991 | 99.10 | 40.00 | 100.43 |
|  | 8.00 | 8.071 | 100.88 | 50.00 | 99.29 |
|  | 10.00 | 9.941 | 99.41 | 60.00 | 100.30 |
| ‾x ± SD |  |  | 99.79 ± 1.21 |  | 100.01 ± 0.62 |
| *t* |  |  | 0.24 |  |  |
| *F* |  |  | 3.76 |  |  |
| Fexon ^®^ tablets  FEX (180.0 mg) | 1.00 | 0.991 | 99.10 | 10.00 | 101.16 |
|  | 8.00 | 8.062 | 100.78 | 20.00 | 98.52 |
|  | 10.00 | 9.952 | 99.52 | 30.00 | 100.54 |
| ‾x ± SD |  |  | 99.77 ± 0.92 |  | 100.07 ± 1.38 |
| *t* |  |  | 0.32 |  |  |
| *F* |  |  | 2.27 |  |  |
| Singulair^®^ tablets  MKT (10.0 mg) | 1.00 | 1.016 | 101.60 | 40.00 | 99.18 |
|  | 8.00 | 7.953 | 99.41 | 50.00 | 101.45 |
|  | 10.00 | 10.036 | 100.36 | 60.00 | 99.35 |
| ‾x ± SD |  |  | 100.46 ± 1.10 |  | 99.99 ± 0.73 |
| *t* |  |  | 0.48 |  |  |
| *F* |  |  | 1.32 |  |  |

*****The tabulated *t* and *F* values are 2.77, 19.00, respectively at p = 0.05 (46)

**Table S2:** Assay results for determination of RUP, DES, FEX and MKT in laboratory prepared tablets by the proposed method

| Compounds | Proposed method | | | | | | | | | | | | | Comparison methods ^(28,31,36)^ | | |
| --- | --- | --- | --- | --- | --- | --- | --- | --- | --- | --- | --- | --- | --- | --- | --- | --- |
|  | Conc. taken  (μg/mL) | | | | | Conc. found  (μg/mL) | | | | | | % Found | | % Found | | |
|  | RUP | MKT | | | | RUP | | | | MKT | | RUP | MKT | RUP | MKT | |
| Lab. prepared tablet of RUP/MKT (1:1) | 1.00 | 1.00 | | | | 1.001 | | | | 1.001 | | 100.10 | 100.10 | 99.12 | 99.68 | |
|  | 8.00 | 8.00 | | | | 7.901 | | | | 8.008 | | 98.76 | 100.10 | 101.13 | 100.54 | |
|  | 10.00 | 10.00 | | | | 10.009 | | | | 9.970 | | 100.09 | 99.70 | 99.58 | 99.77 | |
| ‾x  ± SD |  | | | | | | | | | | | 99.65  ±1.23 | 99.96  ± 1.35 | 99.94  ± 1.05 | 99.99  ± 0.47 | |
| % Error |  | | | | | | | | | | | 0.71 | 0.78 | 0.61 | 0.27 | |
| *t* |  |  |  |  |  |  |  |  |  |  |  | 0.30 | 0.27 |  |  | |
| *F* |  |  |  |  |  |  |  |  |  |  |  | 1.35 | 1.14 |  |  |  |
|  | DES | | MKT | | | | DES | | MKT | | | DES | MKT | DES | MKT | |
| Lab. prepared tablet of DES / MKT (1:2) | 1.00 | | | 2.00 | | | 0.986 | | 2.036 | | | 98.60 | 101.80 | 99.44 | 99.68 | |
|  | 2.00 | | | 4.00 | | | 2.021 | | 3.970 | | | 101.05 | 99.25 | 100.87 | 100.54 | |
|  | 4.00 | | | 8.00 | | | 3.993 | | 8.008 | | | 99.83 | 100.11 | 99.65 | 99.77 | |
| ‾x  ± SD |  | | | | | | | | | | | 99.82  ± 1.23 | 100.38  ± 1.29 | 99.98  ±0.77 | | 99.99  ± 0.47 |
| % Error |  |  |  |  |  |  |  |  |  |  |  | 0.70 | 0.75 | 0.45 | | 0.27 |
| *t* |  |  |  |  |  |  |  |  |  |  |  | 0.19 | 0.48 |  | | |
| *F* |  |  |  |  |  |  |  |  |  |  |  | 2.51 | 7.53 |  |  |  |
|  | MKT | | | | FEX | | | MKT | | | FEX | MKT | FEX | MKT | FEX | |
| Lab. prepared tablet of MKT / FEX (1:12) | 1.00 | | | | 12.00 | | | 1.011 | | | 12.244 | 101.10 | 102.03 | 99.68 | 101.41 | |
|  | 1.50 | | | | 18.00 | | | 1.479 | | | 17.835 | 98.60 | 99.08 | 100.54 | 98.72 | |
|  | 2.00 | | | | 24.00 | | | 2.010 | | | 24.062 | 100.50 | 100.26 | 99.77 | 100.42 | |
| ‾x ± SD |  | | | | | | | | | | | 100.07  ± 1.30 | 100.45  ± 1.48 | 99.99  ± 0.47 | 100.18  ± 1.40 | |
| % Error |  |  |  |  |  |  |  |  |  |  |  | 0.75 | 0.87 | 0.27 | 0.78 | |
| *t* |  |  |  |  |  |  |  |  |  |  |  | 0.08 | 0.23 |  | | |
| *F* |  |  |  |  |  |  |  |  |  |  |  | 7.62 | 1.19 |  |  |  |

*****The tabulated *t* and *F* values are 2.77, 19.00, respectively at p = 0.05 (46)

**Table S****3:** Assessment of greenness of the proposed HPLC method using AGREE metric

| **Criteria** | | **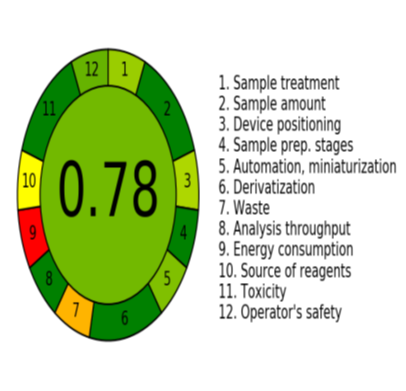** |
| --- | --- | --- |
| 1.Direct analytical techniques should be applied to avoid sample  treatment. | **0.70** |  |
| 2. Minimal sample size and minimal number of samples are goals. | **1.00** |  |
| 3. If possible, measurements should be performed in situ. | **0.66** |  |
| 4**.** Integration of analytical processes and operations saves energy and  reduces the use of reagents. | **1.00** |  |
| **5.** Automated and miniaturized methods should be selected | **0.75** |  |
| **6.** Derivatization should be avoided. | **1.00** |  |
| **7.** Generation of a large volume of analytical waste should be avoided, and proper management of analytical waste should be provided | **0.36** |  |
| **8.** multi-analyte or multi-parameter methods are preferred versus methods using one analyte at a time | **1.00** |  |
| **9.** The use of energy should be minimized | **0.00** |  |
| **10.** Reagents obtained from renewable sources should be preferred | **0.50** |  |
| **11.** Toxic reagents should be eliminated or replaced. | **1.00** |  |
| **12.** Operator's safety should be increased. | **0.80** |  |


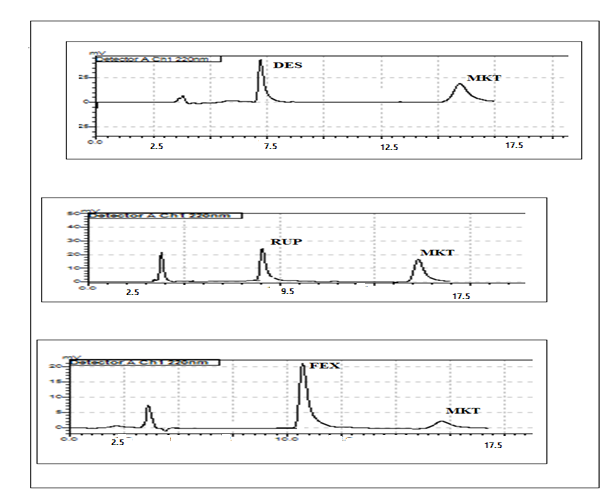


**Fig. S1:** Synthetic mixture of

A: 4 µg/mL of DES and 8 µg/mL of MKT

B: 8 µg/mL of RUP and 8 µg/mL MKT

C: 12 µg/mL of FEX and 1 µg/mL of MKT


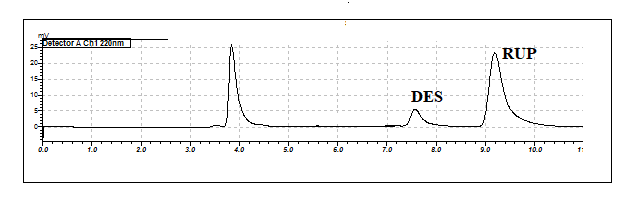


**Fig. S2:**  Synthetic mixture of desloratadine (1 µg/mL) as a degradation product of rupatadine (10 µg/mL)
